# Producing GST-Cbx7 Fusion Proteins from *Escherichia coli*

**DOI:** 10.21769/BioProtoc.2333

**Published:** 2017-06-20

**Authors:** Thao Ngoc Huynh, Xiaojun Ren

**Affiliations:** Department of Chemistry, University of Colorado Denver, Denver, CO, USA

**Keywords:** Polycomb, Cbx7, GST-fusion proteins, pGEX-6P-1, Affinity purification, Glutathione Sepharose 4B

## Abstract

This protocol describes the production of GST-Cbx7 fusion proteins from *E. coli*, originally developed in the recent publication (Zhen *et al.*, 2016). The pGEX-6P-1-GST plasmids encoding the Cbx7 variants were transformed into BL21 competent cells. The fusion protein production was induced by isopropyl-beta-D-thiogalactopyranoside and they were purified by Glutathione Sepharose 4B. This protocol can be adapted for the purification of other proteins.

## [Background]

Polycomb group (PcG) proteins regulate gene expression by modulating higher order chromatin structures (Kerppola, 2009; Simon and Kingston, 2013). PcG proteins are generally found in two major complexes, Polycomb repressive complex (PRC) 1 and 2 (Kerppola, 2009; Simon and Kingston, 2013). PRC2 is a methyltransferase that catalyzes di- and tri-methylation of lysine 27 on histone H3 (H3K27me2/3) (Cao *et al.*, 2002); PRC1 is an ubiquitin ligase that monoubiquitylates histone H2A on lysine 119 (H2AK119Ub) (Wang *et al.*, 2004). Mammalian PRC1 complexes are further divided to canonical and variant PRC1 (Gao *et al.*, 2012, Tavares *et al.*, 2012). Canonical PRC1 is composed of one of each Ring1 (Ring1A/Ring1B), Pcgf (Mel18/Bmi1), Phc (Phc1/2/3), and Cbx (Cbx2/4/6/7/8) proteins. The Cbx family proteins have a conserved chromodomain (CD) that recognizes H3K27me3, suggesting molecular links between the recruitment of canonical PRC1 and H3K27me3 (Blackledge *et al.*, 2015). Recently, we have interrogated the molecular mechanisms underlying the binding of Cbx7-PRC1 to chromatin by live-cell single-molecule imaging (Zhen *et al.*, 2016). We showed that the CD and AT-hook-like (ATL) motif of Cbx7 constitute a functional DNA-binding unit by electrophoretic mobility shift assay (Zhen *et al.*, 2016). Here, detailed conditions are presented which allow the production of GST-Cbx7 fusion proteins from *E. coli*. With modifications, this protocol may be used for the purification of other proteins. The purification of fusion proteins by GST fusion system has been widely applied in various biochemical and structural studies (Harper and Speicher, 2011).

## Materials and Reagents

1. 50 ml conical tube
2. Dialysis membrane (Spectra/Por^®^ Molecularporous membrane tubing, standard RC tubing, MWCO: 3.5 kD) (Spectrum, catalog number: 132720)
3. Bio-Rad Chromatography Column (2.5 × 10 cm Econo-Column) (Bio-Rad Laboratories, catalog number: 7311550)
4. BL21 competent cells (made in the laboratory)
5. pGEX-6P-1-GST (GE Healthcare)
6. Ampicillin
7. Isopropyl-1-thio-β-D-galactopyranoside (IPTG) (Omega Bio-tek, catalog number: AC121)
8. Glutathione Sepharose 4B (GE Healthcare, catalog number: 17075601)
9. Phosphate-buffered saline (PBS, pH 7.4)
10. Pierce™ Coomassie (Bradford) Protein Assay Kit (Thermo Fisher Scientific, Thermo Scientific™, catalog number: 23200)
11. Coomassie Blue (Bio-Rad Laboratories, catalog number: 1610786)
12. Tryptone
13. Yeast extract
14. Sodium chloride (NaCl)
15. 1% Triton X-100
16. Phenylmethanesulfonyl fluoride (PMSF) (Sigma-Aldrich, catalog number: 93482)
17. Protease inhibitor cocktail (Sigma-Aldrich, catalog number: P8340)
18. L-glutathione reduced (Sigma-Aldrich, catalog number: G4251)
19. LB medium (see Recipes)
20. Wash buffer (see Recipes)
21. Lysis buffer (see Recipes)
22. Elution buffer (see Recipes)

### Equipment

1. Culture flasks (2,000 ml) (NALGENE, U.S.A.)
2. Shaker
3. Centrifuges (Eppendorf, model: 5702 R)
4. Centrifuge bottle
5. Vibra-Cell™ sonicator (Sonics & Materials, model: VCX 130) with standard probe (1/4″; 6 mm), length 4.5″ (113 mm), Titanium alloy Ti-6Al-4V, Autoclavable (Sonics & Materials, catalog number: 630-0435)
6. S-3200-2 GyroMixer (BioExpress, model: GeneMate GyroMixer, catalog number: S-3200-2)

### Procedure

*Note: The experimental procedure was revised from the published protocol* (Harper and Speicher, 2011).

**Figure 1:**
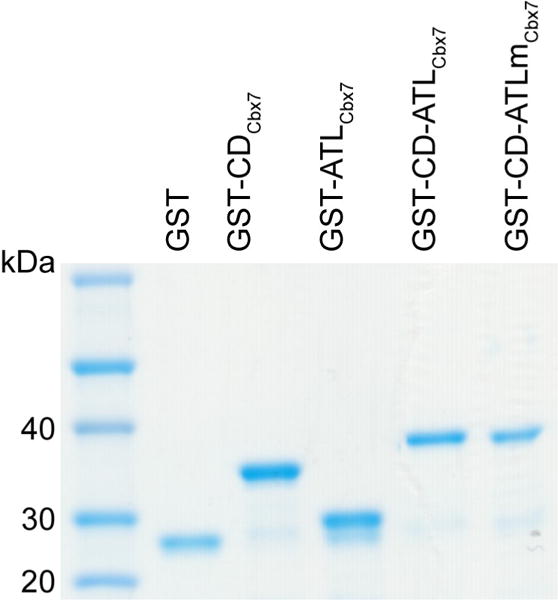
SDS-PAGE gel stained with Coomassie for determination of Cbx7 variant GST-fusion proteins

- Expression of GST fusion protein
  1. Transform competent BL21 cells with pGEX-6P-1-GST plasmids that encode the Cbx7 variants by incubating plasmid with cells on ice for 10 min, then heat shock at 42 °C for 45 sec. The mixture was put on ice for 2 min, LB medium was added, and the mixture was incubated for 1 h at 37 °C while shaking at 250–300 rpm. After that, the cells were spread onto agar plate containing ampicillin.
  2. Transfer a single, isolated colony of transformed BL21 cells to 100 ml LB medium with 100 μg/ml ampicillin and incubate the inoculated culture overnight at 37 °C while shaking at 250–300 rpm.
  3. Transfer 50 ml of the overnight culture into 950 ml of warm, fresh LB medium with 100 μg/ml ampicillin.
  4. Incubate the culture at 37 °C while shaking at 250–300 rpm until the OD600 is 0.5–0.7 (Note 1).
  5. Induce the protein expression by adding IPTG to a final concentration of 1.0 mM (stock concentration: 100 mM, the powder was dissolved in MilliQ water and the solution was filtered before used) and incubating at 37 °C while shaking at 250–300 rpm for 5 h.
  6. Harvest cells by centrifugation at 4,000 × *g* for 20 min at 4 °C.
  7. Carefully decant the supernatant, leaving about 15–50 ml in the centrifuge bottle.
  8. Resuspend the cells and transfer to a 50 ml conical tube and centrifuge at 4,000 × *g* for 20 min at 4 °C.
  9. Decant the supernatant (Note 2).
- Sonication (Note 3)
  1. Resuspend the cell pellet in 25 ml of lysis buffer.
  2. Lyse cells by sonication at 4 °C using the standard probe with the following settings:
    - 15-sec on
    - 45-sec off
    - 45% input (45% amplitude)
    - 6 min: total time on
- Purification
  1. To the mixture after sonication, add Triton X-100 to a final concentration of 1% and mix gently for 30 min at 4 °C with the mixer to increase the solubility of the protein.
  2. Centrifuge the mixture at 10,000 × *g* for 10 min at 4 °C.
  3. Wash 0.75 ml of Glutathione Sepharose beads with 3 × 10 ml of cold PBS, centrifuge at 500 × *g* for 3 min at 4 °C.
  4. Transfer the supernatant by pipetting from step C2 to pre-washed beads in a conical tube and rotate at 4 °C for 1 h.
  5. Centrifuge at 500 × *g* for 3 min at 4 °C and remove the supernatant.
  6. Wash the beads with 4 × 10 ml of wash buffer.
  7. Pour the beads into a Bio-Rad Chromatography Column and wash with 2 × 10 ml of cold PBS.
  8. Elute the fusion protein by incubating at room temperature for 10 min with 1 ml elution buffer. Repeat this step to get a total of three elutions.
  9. Dialyze against PBS three times.
  10. Run SDS-PAGE gel to determine the purity and identity of the fusion protein (Figure 1).

### Data analysis

The Pierce™ Coomassie (Bradford) Protein Assay Kit (Thermo Scientific) was performed to determine the concentration of the protein. SDS-PAGE gel was used to determine the identity of the proteins by their expected molar mass and also to check for contaminates (if yes, there will be other bands shown together with the protein band). Furthermore, GST protein was run with the SDS-PAGE gel as a control. The gel was stained with Coomassie Blue and is presented in Figure 6-figure supplement 1 in (Zhen *et al.*, 2016): Live-cell single-molecule tracking reveals co-recognition of H3K27me3 and DNA targets polycomb Cbx7-PRC1 to chromatin.

### Notes

1. To monitor the OD, measure the absorbance at 600 nm. Estimate the amount of time by assuming the population of *E. coli* doubles every 20 min.
2. The cell pellet from step C10 can be frozen at −80 °C for several months.
3. For sonication: cell disruption is evidenced by partial clearing of the suspension. Avoid over sonication since it will heat the solution, leading to protein aggregation and denaturation.
4. After sonication, 1% Triton X-100 was added, Triton X-100 can be replaced with other detergents such as NP-40 or Tween 20.

### Recipes

1. LB medium (Autoclaved, 1 L, pH 7.2)
  10 g tryptone
  5 g yeast extract
  5 g NaCl
2. Wash buffer
  1× PBS + 1% Triton X-100
3. Lysis buffer
  1× PBS
  0.1 mM PMSF
  0.1 mM protease inhibitor cocktail
4. Elution buffer
  20 mM reduced glutathione, pH 8.0
  Adjust pH with NaOH
